# INCLUSIVE NEIGHBORHOODS TO SUPPORT OUTDOOR MOBILITY AND PARTICIPATION OF PEOPLE LIVING WITH DEMENTIA

**DOI:** 10.1093/geroni/igae098.1332

**Published:** 2024-12-31

**Authors:** Habib Chaudhury, Neil Charness

**Affiliations:** Chair; Discussant

## Abstract

The creation of a supportive and inclusive neighborhood is instrumental to maintaining mobility and social participation of people living with dementia in the community. Achieving this objective requires a clear understanding of how the condition of dementia shapes people’s use of streets and outdoor spaces, and how neighborhood built environmental features influence movement and activity of people living with dementia. This understanding is fairly limited in current research and practice, owing to the paucity of empirical evidence, as well as evidence-based resources that can guide decision-making and planning. These research gaps are addressed in a multi-site, mixed-methods study in British Columbia, Canada, titled “Dementia-inclusive Spaces for Community Access, Participation, and Engagement (DemSCAPE)”. The aims of this study are to 1) explore the outdoor mobility patterns and experiences of people living with dementia in the community, and the role of the neighborhood built environment in shaping their mobility and participation In outdoor activities, and 2) develop evidence-based resources that can guide dementia-friendly and inclusive planning and design. This symposium will also present findings from the knowledge generation and knowledge mobilization phases of the DemSCAPE study. Knowledge generated in the study is based on qualitative and quantitative analyses of data collected with participants living with dementia and care partners in British Columbia. Knowledge mobilization activities involve three key resources developed in our project: documentary video, photo exhibit, and dementia-inclusive planning and design guide. Together, these five presentations offer insights that can guide stakeholders in creating dementia-friendly and inclusive outdoor spaces.

